# Measles outbreak, Montenegro January–July 2018: Lessons learned

**DOI:** 10.1002/jmv.27377

**Published:** 2021-10-11

**Authors:** Boban Mugoša, Giancarlo Ceccarelli, Senad Begić, Danijela Vujošević, Zeljka Zekovic, Massimo Ciccozzi, Zoran Vratnica

**Affiliations:** ^1^ Institute of Public Health of Montenegro Podgorica Montenegro; ^2^ Department of Public Health and Infectious Diseases University of Rome “Sapienza” Rome Italy; ^3^ Migrant and Global Health Research Organization (Mi‐HeRo) Rome Italy; ^4^ Unit of Medical Statistics and Molecular Epidemiology University of Biomedical Campus Rome Italy; ^5^ PZU Diagnostica Podgorica Montenegro

**Keywords:** Balkan, communicable disease, epidemic, Europe, measles, Montenegro, outbreak, vaccine

## Abstract

In 2017, the Regional Verification Commission for Measles and Rubella Elimination (RVC) of the World Health Organization confirmed that measles elimination was sustained in Montenegro, and the previous endemic transmission remained interrupted. However, the RVC was extremely concerned over the continuing low vaccination coverage reported for this country. In this study, we describe the most recent measles epidemic in Montenegro using the epidemiological data collected from January 1 to July 31, 2018. The outbreak is largely attributable to a dangerous accumulation of susceptible subjects across the country and represents a high‐risk factor for re‐establishing endemic transmission in the Balkan area. This study showed how a vaccine‐preventable communicable disease outbreak can have a dramatic impact and severe consequences on regional public health system performance in terms of the sanitary spending point of view. A detailed update is provided on the epidemiological situation in this Central European area, not available until now.

## Highlights

- On 2017, the Regional Verification Commission for Measles and Rubella Elimination (RVC) of World Health Organization confirmed that measles elimination was sustained in Montenegro, and the previous endemic transmission remained interrupted.
- The reduction in vaccination coverage was one of the main causes of the measles outbreak in Montenegro in 2018: this report provides information on the impact of the epidemic on the national health service and complements the gap in epidemiological data on measles trends available for this country of the WHO European Region.

## 1 INTRODUCTION

Measles is among the most contagious viral diseases caused by the measles virus (MeV), a member of the Morbillivirus genus of the Paramyxoviridae family. Indeed, the basic epidemiological reproductive rate (*R*_0_) of measles is much higher in comparison to, for instance, influenza: 12–18 versus 1.4–4^1^, ^2^ reflecting the fact that a single contagious measles case can infect in normal social interaction, on an average, 12–18 people, in a fully susceptible population.

Children are mainly affected in the absence of specific immunity that has been induced by vaccination.

Measles is transmitted via droplets from the nose, mouth, or throat of infected and contagious persons. Initial symptoms, which usually appear 10–12 days following the infection, include high fever, a runny nose, bloodshot eyes, and tiny white spots on the inside of the mouth. Several days later, a rash develops, starting on the face and upper neck and gradually spreading downwards. Severe measles is more likely among poorly nourished young children, especially those with insufficient vitamin A levels, or those whose immune system has been weakened by HIV/AIDS, other diseases, or certain immunomodulatory therapeutic regimes. Serious complications including blindness, encephalitis, severe diarrhea with consequent dehydration, and severe respiratory infections such as bacterial and viral pneumonias, sometimes leading to death, may be due to measles infection.^1^, ^2^, ^3^

Despite the availability of safe and effective vaccines, measles is one of the leading causes of child mortality worldwide, particularly in poor nutrition and inefficient health care systems settings.^4^, ^5^ Following the WHO initiative to eliminate measles by strengthening immunization systems, increasing vaccination rates led to a reduction namely in measles morbidity and also mortality with a 75% reduction in number of measles deaths recorded in the period between 2000 and 2013,^6^ with an estimated 15.6 million deaths prevented in this period.^7^

In communities where vaccination coverage is lower than 95%, outbreaks can easily occur.^8^

In 2010, all 53 countries in the World Health Organization (WHO) European Region (EUR) including Montenegro, reconfirmed eliminating measles and rubella and congenital rubella syndrome as a top political and public health priority^9^ renewing their commitment to achieving those goals with 2015 being set as the new target date for the European regional goals of eliminating measles and rubella. However, and as those efforts have failed, under the Global Vaccine Action Plan (GVAP), measles has been once again targeted for elimination in five WHO Regions by 2020.^10^ WHO is the leading technical agency responsible for the coordination of immunization and surveillance activities supporting all countries to achieve this goal.^11^

**Figure 1:**
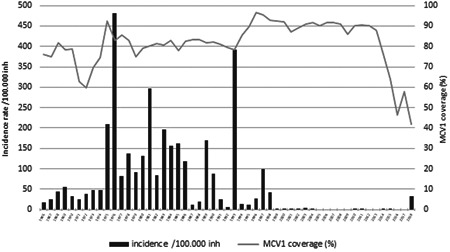
Measles incidence and MCV1 coverage, Montenegro 1966–2018

Before and few years following the introduction of mandatory measles immunization with measles‐containing vaccine (MCV) in Montenegro in 1972 (as mono measles vaccine), measles cases were recorded annually with extensive outbreaks occurring almost every 2–3 years—Figure 1. As a result of the described vaccination strategy and especially following the second dose introduction in 1995 (ever since given as MMR), measles incidence in Montenegro dropped dramatically—Figure 1 (adopted from Annual immunizations coverage report, IPH Montenegro, 2019).

Following five years without cases (2005–2009), measles in Montenegro have been reported in 2010 and 2011 with only five cases confirmed in each of the years. After that period, in September 2014, almost simultaneous importation of four independent cases in four different municipalities resulted in seven locally acquired cases detected by the end of that year. Importation and subsequent small chains of transmission continued during the first 5 months of 2015 with total of 15 cases detected and registered all of which were imported (5) or import‐related (10 cases) in epidemiologically unlinked clusters indicating limited chains of transmission due to favorable herd immunity and high immunization coverage levels. The last measles case in 2015 has been detected during the last week of May and since then, for a period of more than 32 months, no cases of measles have been identified in the country.

Unfortunately and almost exclusively due to extremely declining levels of measles immunization coverage in the last 4 years (2014–2018; Figure 1), and as a result of more prominent anti‐vaccination sentiments in whole of the region, as well as the fake news, in 2018, mostly due to several importations mainly from neighboring Serbia, Montenegro has experienced clusters of measles cases resulting in a nationwide outbreak with 200 cases (179 laboratory‐confirmed, 4 epidemiologically linked, and 17 clinically compatible cases) with measles incidence rate in 2018 of 292.4 per 1 million population.

In total, 275 cases were epidemiologically processed as measles‐like cases with the rate of discarded cases being 12.1.

At the same time, a total of 41 000 measles cases, including 37 deaths occurred in the first 6 months of 2018, in seven European countries highlighting measles as a European problem.^12^ In total, and as of December 10, 2020, 89 148 measles cases have been registered in the European WHO region during 2018.^13^

In this study, we describe the most recent measles epidemic in Montenegro using the epidemiological data collected from January 1 to July 31, 2018.

## 2 METHODS

Mandatory notification of measles in Montenegro with comprehensive population coverage has been in place since the late sixties. Nevertheless, and to improve case detection and increase the specificity of the surveillance system—mandatory reporting has also been imposed on microbiology laboratories along with microbiological investigation of every single case with clinical presentation of rash and fever ever since 2010.

Both clinicians and microbiologists are requested to report suspected, investigated, and confirmed measles cases immediately to the local epidemiology service as well as to the Institute for Public Health (IPH)—the institution responsible for coordinating and implementing surveillance and control measures on the national level.

Regarding the immunization policies—mandatory measles vaccination with a single monovalent dose has been introduced in 1972 targeting all children in the second year of life. The monovalent vaccine has been later changed to combined measles‐mumps (MM or in local language “Mo‐Par”) vaccine only to be replaced in 1995 with a combined measles‐mumps‐rubella (MMR) vaccine with an additional dose of the vaccine introduced in a prescribed schedule for children aged 12 years. Following a couple of years of implementation and based on the observed epidemiological data and age of the cases, the second dose has been shifted to preschool‐age children (6–7 years) and is still currently given at that age—before the enrolment in primary schools.^14^

## 3 CASE DEFINITIONS AND DATA SOURCE

Classification of measles cases has been done according to the WHO definition and criteria.^15^ Laboratory confirmation of measles has been performed in the National WHO Referent Laboratory at the Unit of Virology within the IPH. Measles surveillance data—number of notifications and samples, between January 1 and December 31, 2018, including measles vaccine coverage data during the same period, have been obtained from the surveillance database of the IPH and National immunizations registry that is also run by the IPH. Database, diseases under surveillance, its definitions, and the manner of reporting are fully aligned with European union legal acquis and European Commission decision from 2012.^16^

The annual incidence rates of measles were measured per 100 000 inhabitants. The numerator was the number of the measles cases (compatible, laboratory‐confirmed, and epidemiologically linked) in the total population of Montenegro while the denominator was the whole population monitored during 2018.

To estimate the annual measles immunization coverage rates in Montenegro, the total number of immunized children (numerator) within one calendar year was divided by the total number of children who should have been immunized according to their age or year of birth by Montenegrin immunization schedule (denominator). The data on immunization coverage from immunization records of children were obtained as a part of routine surveillance of mandatory immunization in Montenegro.^14^

### 3.1 Statistical analysis

Parametric and nonparametric statistical tests have been applied including the Chi‐square test, to evaluate possible differences of certain attributes between measles cases who have been hospitalized versus those who were not treated in hospitals. Logistic regression has been used to identify variables predicting hospitalization; only variables associated with the hospitalization statistically significant in the univariate analysis have been considered suitable for multivariate analysis. A *p*‐value lower than 5% has been considered statistically significant. The analysis has been performed using STATA V.14.

### 3.2 Ethical consideration

The study has been done in the framework of public health surveillance on communicable diseases in Montenegro. Sample and data collection was part of the standard patient and public health management of suspected measles cases and required oral informed patient consent. Access to identifiable patient data has been restricted and allowed only to IPH employees who have been directly involved in measles surveillance and diagnosis in accordance with the national legal framework.

## 4 RESULTS

**Figure 2:**
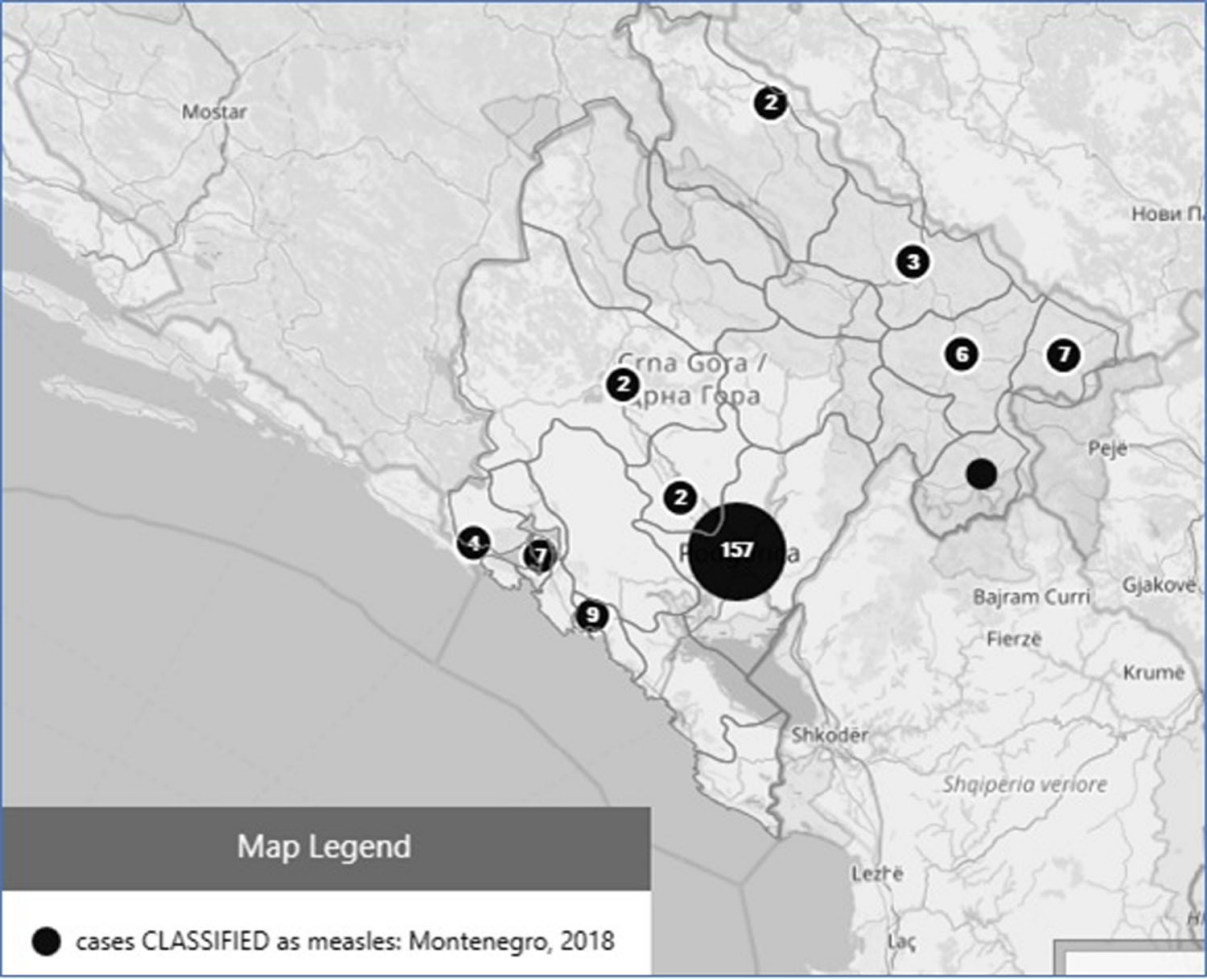
Map of geographical distribution of measles cases, Montenegro 2018. Size of circle reflects the number of cases

In 2018, a total number of 272 patients with suspected measles (suspected case = laboratory‐confirmed case + clinical case) have been reported in Montenegro out of whom 180 (71.43%) have been hospitalized (Figure 2). Males accounted for 49.63% with the median age of 12.5 years (1–30) among all suspected, and 5.5 (1–28.5) and 20.5 (3–32.5) years among hospitalized and nonhospitalized cases, respectively. This difference has been statistically significant (*p* = 0.017).

As expected in epidemic situation, 78.3% of all suspected cases have not been vaccinated. A significantly larger proportion of cases who have been hospitalized received at least one dose of vaccine (44.19%) as compared with patients who have not been hospitalized (14.04%, *p* < 0.001). The differences between hospitalized and not hospitalized patients, in number of measles vaccines, have been statistically significant probably due to low vaccination rates and small numbers rather than the number of doses themselves.

Among patients who received the measles vaccine, no difference in time from vaccine application to rash onset has been found between hospitalized and nonhospitalized patients. Furthermore, higher prevalence of complications, laboratory confirmations of the disease, and final classification diagnostic for measles have been reported in patients who have been hospitalized as compared with patients who have not been hospitalized.

About 25% of patients did not actually have received a confirmation of measles suspicion by laboratory testing and clinical follow‐up. Three patients have been lost during the follow‐up (two were hospitalized) and no deaths have been reported (Table 1).

**Table 1:** Demographic and epidemiological characteristics of the study population

	All suspected cases	Hospitalized (*n* = 180)	Non hospitalized (*n* = 92)	*P*‐value of hospitalized versus nonhospitalized
Sex ratio: M/F (%)	49.63/50.37	54.17/45.83	50.56/49.44	0.68
Median age, years (IC range)	12.5 (1–30)	5.5 (1–28.5)	20.5 (3–32.5)	0.017
Measles vaccination, N/Y age years?	78.31/21.69	55.81/44.19	85.96/14.04	<0.001
Number of vaccines 0/1/2 (%) doses	78.31/9.04/12.65	55.81/18.60/25.58	85.96/6.14/7.89	<0.001
Median time in years – from last vaccination to rash onset (IC range)	9.435 (2.34–17.68)	6.85 (2.79–14.85)	10.22 (2.34–18.53)	0.23
*Source of infection (%)*				0.002
Imported	5.51	7.78	0	
Not imported	31.62	34.44	25	
Import related	2.57	3.89	0	
Unknown	60.29	53.89	75.00	
Complications Y/N	85.55/14.45	80.72/19.28	92.86/7.14	0.019
Encephalitis Y/N	1.47/98.53	2.22/97.78	0/100	0.6
Pneumonia Y/N	4.30/95.7	5.42/94.58	2.86/97.14	0.5
*Final classification (%)*				<0.001
Discarded as measles	25.75	19.21	43.66	
Measles LAB confirmed	65.67	71.75	47.89	
Measles EPID linked	1.49	0.56	2.82	
Measles vaccine‐related	7.09	8.47	5.63	
*Final classification*				<0.001
Measles not confirmed	25.75	19.21	43.66	
Measles confirmed	74.25	80.79	56.34	
*IgM*				0.006
Not tested	21.69	18.33	26.39	
Positive	51.47	58.33	34.72	
Negative	22.43	19.44	31.94	
Inconclusive	4.41	3.89	6.94	
*Virus isolation*				0.15
Not tested	72.79	75.00	66.67	
Positive	21.69	21.11	23.61	
Negative	5.51	3.89	9.72	

In multivariate analysis, the ORs have been found statistically significant for age (0.98 [CI; 0.95–0.99], *p* = 0.02) and presence of complications (3.03 [CI; 1.1–8.4], *p* = 0.033; Table 2).

**Table 2:** Univariate and multivariate analysis

	Univariate		Multivariate	
	OR and OR interval	*p*	OR and OR interval	*p*
Female sex	1.155844 (0.6682119–1.99932)	0.6		
Age	0.9817865 (0.9648115–0.9990601)	0.039	0.9772677 (0.9584695–0.9964345)	0.02
Number of vaccinations	0.9671952 (0.9072096–1.031147)	0.3		
Source of infection (not imported vs. imported)	1.917526 (1.030099–3.569468)	0.04	1.885012 (0.977805–3.633924)	0.058
Complications	3.104478 (1.155884–8.338017)	0.025	3.038013 (1.095468–8.425189)	0.033

## 5 DISCUSSION

Although more and more children in the WHO European Region are being vaccinated against measles, the progress achieved has not been sufficient across the countries with huge differences among, and even within, specific countries thus leaving the clusters of susceptible individuals unprotected and resulting in a record number of people affected by the virus in 2017, 2018, and 2019, even in the most developed countries of the European Union.^16^, ^17^

Furthermore, the number of the deceased reached a record high in the last few decades (72 children and adults dead in the WHO European Region alone in 2018).

Despite the availability of an effective and safe vaccine, the measles outbreak across the continent, as well as globally, continues to put a strain on health care systems, with outbreaks occurring in areas and populations with suboptimal immunization rates. Moreover, as two doses of the vaccine have been recommended to ensure full and adequate immunity, a significant proportion of vaccinated subjects have received only the first dose.

A statistically significant difference between hospitalized and not hospitalized patients in terms of number of measles vaccines given has been probably due to low vaccination rates and small numbers rather than the number of doses themselves. At the same time, this finding can also be explained by the age of hospitalized patients that has been significantly lower respected than the nonhospitalized patients. Also, it is expected in the general population to have a higher compliance to vaccination programs in childhood than in older age. Cases were also observed among fully vaccinated which may be a consequence of the waning immunity over time or certain (more than expected) proportion of vaccinated children who failed to develop immunity from the first dose with the implication that the real vaccine‐induced immunity is probably much lower than expected and significantly lower than the number of persons vaccinated. Nevertheless, these findings should be further investigated.

The main circulating genotype reported in Montenegro was B3, pretty much the same as observed in other European countries at that time.^18^, ^19^

Interestingly, the majority of cases observed in this outbreak were in pediatric patients. This suggests that the adult population was adequately protected, probably as a result of both vaccination programs implemented in previous years and measles epidemics recorded in the Balkan area in past years, similarly to what has been observed also in other countries.^20^ In particular, measles vaccination was introduced in the Socialist Federal Republic of Yugoslavia) in 1971 as monovalent vaccine administered at pediatric age in single dose, and mandatory mass measles vaccination started in 1972. In 1993, the monovalent vaccine was replaced by the two‐dose MMR vaccine.^21^, ^22^ Moreover, live measles vaccine prepared from a further‐attenuated Edmonston‐Zagreb strain was also used for vaccination in Yugoslavia.^23^ Before the introduction of measles immunization in 1971, large measles outbreaks in Yugoslavia were recorded every 1–3 years, mainly with cases reported among preschool children. In the postvaccination era, the measles incidence dropped dramatically, and only a small measles outbreak were reported, probably attesting to a good level of overall vaccination coverage in the general population at least until the beginning of the Yugoslav Wars fought from 1991 to 2001.^24^ In the post‐war period, a number of epidemiologically significant measles outbreaks have been described in the Balkan area especially in Serbia and Macedonia between 2007 and 2011, testifying to a possible decrease in overall vaccination coverage partly due to both the fragility of local health systems and massive population movements following the war crisis.^25^, ^26^ All these data could explain the low prevalence of contagions in adulthood during the outbreak described in Montenegro.

This study gave a snapshot of the measles surveillance results in 2018 to understand the real public health repercussions of a highly contagious vaccine‐preventable disease during an outbreak in specific settings including nonmedical circumstances and huge general public and media interest.

The high proportion of hospitalized suspected cases could suggest that many of the hospitalizations were not medically indicated and were rather performed either as a control measure implemented by general practitioners and pediatricians working on primary level of health care, or out of the huge public interest and media coverage of the outbreak and severely high mortality rates observed in the neighboring countries—Serbia 14, Italy 9, and Albania 3.^27^, ^28^

By our experience and although the vaccination against measles is free of charge and mandatory,^29^ in the last few years, there has been a trend of decline in immunization rates mainly for the first dose of MCV in Montenegro. The situation lead to a measles outbreak with a different age‐specific distribution involved, mostly affecting younger, with statistically significant difference in age between hospitalized and nonhospitalized cases, as expected.

A decreased immunization has been most likely due to skepticism toward vaccination, fake news, and negative immunization messages shared on social media among inexpert people and probably mirroring the effect of an increased anti‐vaccination movement in several European countries.^30^

## 6 CONCLUSIONS

Our study showed how a vaccine‐preventable communicable disease outbreak can have a dramatic impact and severe consequences on regional public health system performance in terms of sanitary and public health spending point of view.^31^, ^32^ The Montenegrin measles outbreak in 2018, has been the consequence of a suboptimal vaccination coverage among children and insufficient catch‐up immunization campaigns among younger adults, resulting in an accumulation of measles susceptible and prone individuals.

Although the availability of rapid diagnostic tests may facilitate measles management and response, vaccination still remains the best possible way to prevent outbreaks and their potential severe impact on population as of whole.^33^ Therefore, there is an urgent need and utmost public health imperative to improve vaccination coverage rates with both doses of MMR vaccine and especially with the first dose among children aged 12–15 months and then and young adults both in the general and hard‐to‐reach, mobile, populations.

Although this study did not focus on the probable source and place of exposure, prevention of measles transmission in healthcare institutions should also be strengthened.^31^

## AUTHOR CONTRIBUTIONS

Boban Mugoša designed the study and wrote the manuscript. Senad Begić, Danijela Vujošević, and Zeljka Zekovic acquired and analyzed data, contributed to the evaluation of the results. Giancarlo Ceccarelli contributed to the evaluation of the results and to the discussion, critically revised the manuscript. Massimo Ciccozzi and Zoran Vratnica designed the study and critically revised the manuscript.

## CONFLICT OF INTERESTS

The authors declare that there are no conflict of interests.

## Data Availability

The data that support the findings of this study are available on request from the corresponding author. The data are not publicly available due to privacy or ethical restrictions as the National communicable diseases surveillance database is not routinely anonymized.
